# A rare case of double J stent migration in the kidney

**DOI:** 10.1016/j.eucr.2020.101557

**Published:** 2021-01-06

**Authors:** V. Dunev, P. Genov, V. Mladenov, P. Antonov, B. Atanasov

**Affiliations:** a Medical University Pleven, “Georgi Kochev”8A str, 5800, Bulgaria; b University of Ruse “Angel Kanchev”, Ruse, 8 “Studentska” str, 7000, Bulgaria; c Medical University Sofia, 1 “Georgi Sofiiski” str, 1431, Bulgaria; d Medical University Plovdiv, 15 “Vasil Aprilov” str, 4002, Bulgaria

**Keywords:** Double J stent, Complications, Migration of the double J stent

## Abstract

Double-J stents are among the basic and commonly used tools in urology. There are a lot of complications that can occur during DJ placement. We are presenting 62 years old woman, who was admitted in Urology Department with symptoms of left lumbar pain, irradiating towards inguinal area and hematuria. The computed tomography (CT) scan of abdomen and pelvis defined a propagated DJ stent in the left kidney. Lumenis Holmium laser VersaPulse 100 W was used for resection of the double J stent and after that it was removed from the kidney in pieces.

## Introduction

Double-J (DJ) stents are among the basic and commonly used tools in urology. There are a lot of complications that can occur during DJ placement which may be minor in form like hematuria, dysuria, frequency, flank and suprapubic pain to major complications such as vesicoureteric reflux, migration, malposition, encrustation, stent fracture and etc. They need to be replaced or removed within 6 weeks to 6 months to avoid complications like encrustations, stone formation, fractures and blockades of stents.

## Case Presentation

**Fig. 1:**
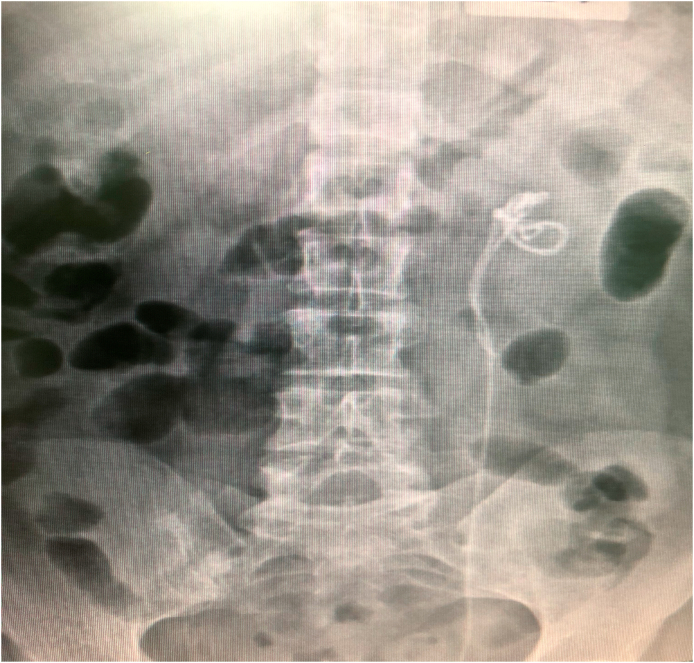
X-ray of the left kidney with two double J stents placed in it.

We are presenting 62 years old woman, who was admitted in Urology Department with symptoms of left lumbar pain, irradiating towards inguinal area and hematuria. The patient had been underwent a double J placement after ureter stone extraction in another department before one week. The computed tomography (CT) scan of abdomen and pelvis defined a propagated DJ stent in the left kidney with distal end in the proximal ureter. We performed ureteroscopy with attempt to extract the stent, but a simple extraction was impossible and the ureteroscope could not pass through the uretero-pelvic junction (UPJ). The decision of putting a second DJ stent was made and performed to ensure unobstructed drainage of the kidney (Fig. 1).

**Fig. 2:**
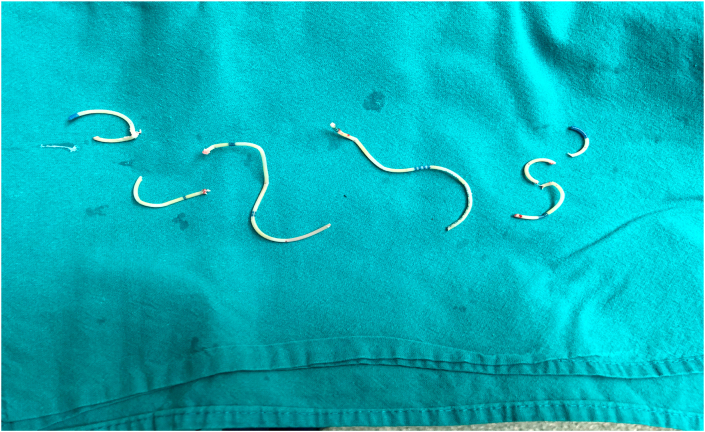
Removed double J stent in pieces.

After 1 month a new ureteroscopy was performed and this time the UPJ was passed with semi-rigid ureteroscope. Entering into the renal pelvis a double J tied in a knot was visualized and it was obvious that it cannot be removed whole from the kidney. Lumenis Holmium laser VersaPulse 100 W was used for resection of the double J stent and after that it was removed from the kidney in pieces (Fig. 2). The postoperative period passed without complications and the patient was discharged on the second day after surgery.

## Discussion

Since its introduction in 1967 by Zimskind et al. DJ stents placement has become routine in the management of a variety of urinary tract disease processes. It is used as an important adjunct to many urologic procedures such as uretero-renoscopy with lithotripsy (URSL), percutaneous nephrolitholapacsy (PCNL) and pyeloplasty. Ureteral stents may also be useful for managing conditions such as hydronephrosis due to a malignant neoplasm, pyonephrosis and obstructive uropathy. The indications for stent insertion have increased and the patients presenting with complications of stent have become more frequent.^1^^,^^2^ The complication in our case was propagation of the double J stent in the kidney.

One of the early comlications of double J placement is a migration of the stent within the urinary tract. Gibbons et al. initially addressed the problem of downward migration of soft silicone tubing by adding barbs along the shaft of the tube, as a result there is a stent design that bears his name. Nevertheless, peristalsis may discharge a stent (especially one constructed from softer materials) from the ureter.^3^ Migration upward or downward can also occur as a result of late reconstitution of the retention curves.^4^ In our case the suggestion for propagation in the ureter was incorrect double J stent placemenet.

## Conclusions

Although the placement of a double J stent is a routine procedure in the modern urological practice, the good knowledge of possible complication of its placement is the key to successfully dealing with these possible complications.

## Declaration of competing interest

The authors declare that they have no competing interests.
